# Supplementary material for: Latent class analysis of substance use typologies associated with mental and sexual health outcomes among sexual and gender minority youth
Source: PLoS One. 2023 Sep 28;18(9):e0290781. doi: 10.1371/journal.pone.0290781 (PMC10538794; doi:10.1371/journal.pone.0290781)
Supplement: Attachment — Submitted filename: tara revision.docx [file pone.0290781.s002.docx]

**Reviewer #1:** COMMENTS TO AUTHORS:
Overall comment: Thank for the opportunity to review this manuscript as it in an important area of work. However, there are a few issues with this paper that needs to be addressed. The paper also needs editing and proof-reading before submission.
Abstract
1. The statement “We applied latent class analysis…. (line 30-33)” needs rewording. In its current form it reads values on substance use variables were used to create latent class to predict substance use patterns. Please revise this.

*Thank you for this comment. The authors agree that this was unclear and have added that multinomial logistic regression was used to investigate which factors were associated with substance patterns.*

2. There are two “=” signs when mentioning SD values.
Introduction

*One of these signs has been deleted.*

1. The introduction needs some work, and focus. The second line in first para (line 49, page 3), please ensure you are using the correct in-text citation when referencing to CDC surveillance, and rather than stating it as ‘recent’, please provide the year.

*The year that this report was published is now included.*

2. Similarly, please provide the year for YRBS survey (line 54, page 3).

*This was data that was collected over a number of years, which was clearly stated in the text on page 3.*

3. There is also inconsistency in which the target population has been described throughout the paper; namely, young men who have sex with men (YMSM), sexual minority youth, sexual and gender minority, sexual and gender minority youth who have sex with men, sample of young men (cisgender and gender non-conforming), groups of youth, etc. Please revise the manuscript, and check for these inconsistencies. If it helps, operationalize it in the start of the paper, and explain what it means for this project.

*The authors agree that these terms have been used inconsistently, partly due to the change in language in literature and recommended for non-stigmatizing language over the previous few years. This has now been consistently changed to represent sexual and gender minority youth, which encompasses the sample that was recruited into this study. This term has been operationalized at the start of the background section, and then the abbreviation used going forward (SGMY). If previous literature refers to sexual minority youth, or gender minority youth only, this term is separated and the acronyms SMY and GMY are used accordingly.*

4. I think the authors meant multiple forms or types of discrimination and not multiple discrimination indicating count of discrimination (line 66, page 3).

*This change is indeed correct and this sentence has now been modified accordingly.*

5. Page 4 (lines 70-77), it would be advisable to refer to minority stress theory/framework (Meyer, 2003), to contextualize the distal stressors as underlying mechanisms. It would also help to bind this para.

*The minority stress theory was previously introduced in the discussion section, but the authors are in agreement that this does need to be introduced earlier. It is now included in the background and fleshed out slightly.*

6. The references on specific STI’s are not needed (line 78, page 4), rather use references/citations on variables that are actually used in this paper. Otherwise, it sets readers to expect findings associated with STIs.

*These references have now been deleted, as per the reviewer’s suggestion.*

7. Page 5, line 95. Please revise the aim, it reads the purpose of this study is to better inform strategies; however, that is what is expected to happen from the results, but is not the main aim (and scope of this study).

*The aim has been slightly revised, and this has rather been suggested as an area for future studies in the discussion section.*

Methods
1. Page 5, 101, again, the sample is referred to with another phrase “sample of young men who have sex with men, some of whom also identified as transgender or non-binary”. Please ensure consistency.

*Again, the authors would like to thank the reviewers for noticing the inconsistency in terminology and have conducted a global search to ensure that the way the included sample is referred to is consistent and to avoid any confusion for the reader.*

2. Page 7, line 137: authors have used ‘racial categories’ and ‘racial and ethnic categories’ in the same para. Please consistently use terms.

*The authors appreciate this observation and have made the necessary corrections throughout the paper to use the terms "racial and ethnic categories."*

3. The methods section doesn’t state sample size for the study, for this particular paper (if not used the entire sample enrolled in this project).

*This is an important point, and the authors have revised this section in the methods that explains the sample size accordingly. Then sentence reads, “We utilized baseline data (n=414) from a 4-arm factorial randomized controlled trial to evaluate the effectiveness of a brief intervention targeting substance use reduction and increased engagement in HIV prevention…”*

4. The methods section also lacks a discussion on missingness in the variables and how missingness was handled in subsequent analyses.

*Thank you for this valuable input regarding the treatment of missing data. The authors have taken this comment into consideration and included a description of the missing data handling approach that was used in the methods section to address this point. The sentence reads, “In Mplus, missing data is handled using a technique called full information maximum likelihood (FIML) estimation. Mplus leverages FIML estimation to optimize the utilization of available data and generate robust estimates of model parameters, even in the presence of missing data points.”*

Results/ Discussion/ Limitations
1. Can authors please clarify why a 4-class solution was selected (as stated by authors, BIC was higher for class 4 compared to class 3). I would also suggest adding -2Loglikelihhood and adjusted BIC (aBIC) values to the table. More importantly, my issue with the class selection is with the sample size in each class. Class 1 and class 2 have less than 5% of sample (n=14 (3.4%); and n=19 (4.6%); respectively). Classes 3 and 4, combined account for 92% of the sample. Most research suggests, to have at least 5% of the sample in each class; and with the smaller sample size in general, can authors clarify and discuss if the LCA regression analyses had enough power to detect differences.

*The authors acknowledge this valuable comment about the decision to include a 4-class vs a 3-class model, and this is something that has been carefully considered. We have identified several compelling reasons to proceed with the 4-class model, and have also expanded on this reasoning in the manuscript. The reasons are as follows:*

1. *Fit statistics. We have incorporated the adjusted Bayesian Information Criterion (aBIC) and -2loglikelihoo values in Table 1. After accounting for sample size, we observed that the BIC was lower for the 4-class solution compared to the 3-class solution. Additionally, the -2loglikelihood followed a similar pattern, decreasing as the number of classes increased. Hence, we could conclude with 4-class model with the fit statistics.*
2. *Membership probability. Moreover, it is crucial to highlight that the average estimated posterior class-membership probabilities for all four classes exceeded 0.87, suggesting strong membership in their respective classes. Notably, the probabilities for class 1 and class 2 were particularly high, surpassing 0.93.*
3. *Interpretability. The decision to retain the 4-class model was also made based on the aims of the specific manuscript and the patterns of substance use in these classes being different enough to make meaningful interpretations of the classes in the results and discussion section. This is further supported by the significant differences in mental health and other factors which are associated with the substance use typologies in the multinomial logistic regression.*
4. *Class size cannot be the only solution. Upon running the 3-class model, we observed that it also contained a class with a sample size smaller than 5% of the total sample. In light of this consideration, we could not make our decision solely based on class size. Instead, we followed Geiser's recommendation (Geiser, 2011, Datenanalyse mit MPlus) to prioritize interpretability and goodness-of-fit measures when selecting the final class solution.*

*While we believe that our decision was the most appropriate decision, we do recognize the limitation posed by smaller class sizes and the resulting lack of statistical power. We have taken this into consideration and thoroughly discussed this in the limitation section of the discussion.*

2. The confidence intervals for some results are too wide, namely, in the unadjusted modes (page 13, line 269-276). Can authors please comment on this; and clarify this is not due to the power issue (enough samples in each class).

*We acknowledged the power issue related with unequal class sizes, and have thoroughly addressed this issue in the limitation section on Page 19. The sentence reads, “Class prevalence were unequal, with low sample sizes in Classes 1 and 2. This unequal class sizes adversely affected the precision of our estimates, consequently leading to wider confidence intervals. Consequently, the generalizability of our results is limited, and the study's power is reduced. However, it is important to note that our decision was not solely based on class size. Instead, we followed Geiser's recommendation (Geiser, 2011, Datenanalyse mit MPlus) to prioritize interpretability and goodness-of-fit measures when selecting the final class solution.*

3. The discussion section sufficiently describes the findings and contextualizes these in the literature; however, it needs a focus and re-organizing. The authors should focus on discussing the major findings on key variables; and limit the discussion on control variables.

*Thank you for this thoughtful comment which has resulted in the first two paragraphs being slightly reworked. However, we have also been guided by the format of previous studies that have used LCA for substance use typologies, which have also looked at factors associated with classes of substance use.*

4. The authors may refer back to minority stress theory in the discussion, to discuss the findings.

*The minority stress framework has been included in the background, and also been expanded upon in the discussion section.*

5. If intervention strategies are one of the aims of this paper, then the authors must include a separate section, discuss what we currently have, and where interventions need to be adapted, or new interventions need to be developed.

*This has been revised in the manuscript aims, so that is now rather a recommendation for future research which appears in the discussion based on the results.*

6. Including, class sample proportions in the limitation section is not enough, the authors need to address this in the methods/results section; and even consider a 3-class solution.

*Once again, it is worth noting that the 3-class solution did not outperform the 4-class solution in terms of the fit statistics we examined. Furthermore, the 3-class solution also included a class with a sample size smaller than the 5%. However, we acknowledge and have thoroughly addressed the limitation associated with small class sizes as mentioned above on Page 19.*

**Reviewer #2:** COMMENTS TO AUTHORS

Thank you for the opportunity to review this manuscript, which uses an LCA to determine typologies of substance use in YMSM as well as correlates of class membership. The article is interesting with good subject matter. I have a comments and suggested edits, more based on grammar than substance:

Line 49- “The recent HIV Behavior,” “The” should not be capitalized. Also, I believe it is called the “National HIV Behavioral Surveillance”

*This has been revised and is now on line 52 based on previous changes made.*

Line 61- I find this sentence a bit confusing: “who have also been found to have up to four times substance higher use than cisgender youth.” Do you mean they use substances at a rate 4 times higher than their cisgender peers? Or 4 times the substances? Please clarify.

*The authors thank the reviewer for the request to clarify this sentence, which has been done on line 65 to indicate that this was with reference to an increased prevalence of substance use.*

Line 81- “Studies have found also found,” please edit to avoid duplicate language.

*This has been edited, and the entire manuscript has been checked for similar grammatical and/or spelling errors.*

Line 95- There are a few Detroit’s in the US. It would be helpful to clarify that this is Detroit, Michigan.

*This has been clarified in all places where Detroit or Michigan is referred to in the manuscript.*

Line 101- Sometimes you use YMSM, other times you spell it out. It’s good to be consistent and only use YMSM after you’ve defined your acronym the first time.

*The authors agree, and have revised the terminology used throughout the manuscript to ensure consistency, and refer to the sample now as sexual and gender minority youth, after explaining this the first time it is used at the beginning of the background section.*

Line 142- “previous abuse by police” is interesting. How did you define that?

*A definition of abuse by police according to the questions asked in the baseline instrument has been added on page 9.*

Line 174- “one third” should have a hyphen (one-third). So should two-thirds on line 176

*Both of these changes have been made on Page 9.*

Line 177- Please include a % for 63.8

*The authors appreciate the attention to detail in this review, and have made this change on Page 9.*

Line 181- You have already defined PrEP on line 145, please only use the acronym from now on.

*Only the acronym is used after the full definition of the term.*

Line 262, 266- When you talk about controlling for other factors, what specific factors are you controlling for?
Thank you for your feedback. We have incorporated a sentence into the methods section to provide an explanation of the adjusted model. The sentence reads, “*The adjusted multinomial logistic regression models consist of all the variables used in the bivariate multinomial logistic regression models, except for the racial and ethnic categories*.” Furthermore, we have enhanced the clarity of the sentence by modifying it to "controlling for other factors in the unadjusted models."

Line 305- Just a question for consideration, if you were looking at any cannabis in the last 3 months, but hazardous alcohol use, do you have any data on the prevalence of participants that may have hazardous cannabis use (CUDIT)? To me it seems any cannabis use vs. hazardous use if very different, same as any alcohol vs. hazardous alcohol use.

*Thank you for your feedback. In our study, we utilized the ASSIST and AUDIT scores to collect data on alcohol use, while relying solely on the ASSIST for capturing information on other current drug use (ie use in the previous three months), including cannabis use. Consequently, the only measure we could extract regarding cannabis use was from the ASSIST. However, we were unable to utilize the ASSIST for current alcohol use due to a lack of variability, as 92% of participants reported alcohol consumption within the past three months. To address this limitation, we assessed hazardous alcohol use using the AUDIT score, especially since the time period of measurement differed.*

Line 308- were typologies compared to gender identity overall or transgender identify specifically?

*This has been modified to read as gender identify in the first paragraph of the discussion.*

Line 324- “In two drug classes, opioid use was relatively high.” Please name the classes for reader clarity, otherwise they have to go back and search.

*These have been clarified as Class 1 and 2: depressant and stimulant use, and high polysubstance use respectively to ensure that these classes are easily identified.*

Line 325- I find this sentence confusing, “together overdose risk that is especially pertinent with YMSM
youth that belong to who may face barriers to harm reduction and treatment services.” First, YMSM are youth so this is redundant. Second, what do you mean “youth that belong to who may face barriers”? I believe this sentence could use some edits for clarity.

The terminology has been changed in the as well as

Line 331- I find this sentence confusing, “The increased risk of opioid use among been explained within the minority stress framework.” I believe some edits could help to clarify meaning.
